# Robotic tracheobronchoplasty with vertical suturing for excessive dynamic airway collapse

**DOI:** 10.1016/j.xjtc.2025.03.024

**Published:** 2025-04-09

**Authors:** Alessio Campisi, Hauke Winter, Claus Peter Heussel, Martin E. Eichhorn

**Affiliations:** a Thoracic Surgery Unit, Cardiovascular and Thoracic Department, University and Hospital Trust-Ospedale Borgo Trento, Verona, Italy; b Department of Thoracic Surgery, Thoraxklinik, Heidelberg University, Heidelberg, Germany; c Translational Lung Research Center Heidelberg, Member of the German Center for Lung Research, Heidelberg, Germany; d Department of Diagnostic and Interventional Radiology with Nuclear Medicine, Thoraxklinik, Heidelberg University, Heidelberg, Germany

**Figure:**
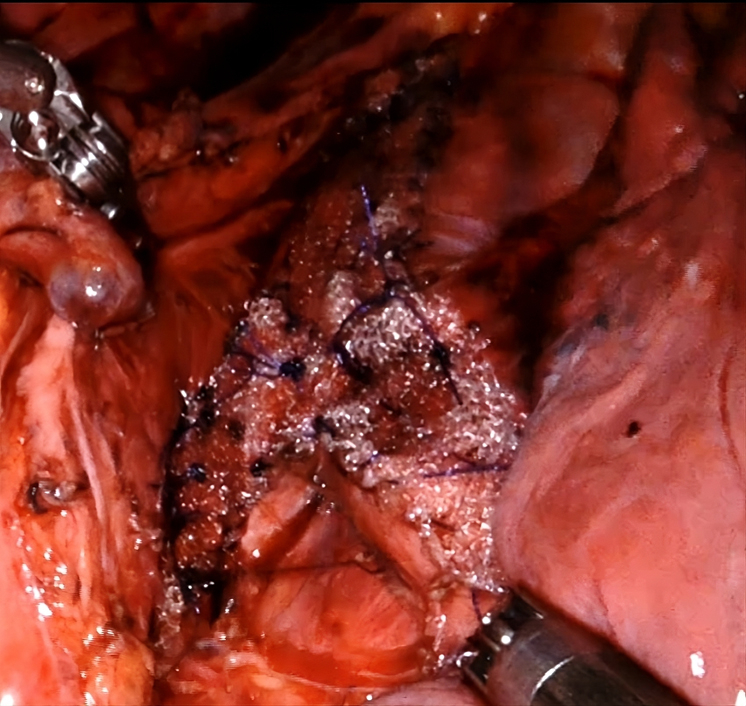
Intraoperative view of robotic tracheobronchoplasty with mesh-reinforced posterior wall.

**Central Message**

Robotic-assisted tracheobronchoplasty with vertical suturing may simplify the process, reducing operative time while ensuring stability.

Excessive dynamic airway collapse (EDAC) is a significant cause of airway obstruction resulting from excessive bulging of the posterior tracheal membranous wall into the lumen during expiration.^1^ Nonsurgical interventions, such as continuous positive airway pressure therapy and endobronchial stenting, can provide temporary relief but do not address the underlying structural abnormalities of EDAC. Tracheobronchoplasty (TBP) remains the definitive intervention for severe cases.^2^ Nevertheless, due to the controversy regarding the benefits of airway stabilization for EDAC, the decision to perform TBP should be made following a thorough multidisciplinary discussion, considering patient-specific factors and the failure of conservative measures. In recent years, minimally invasive approaches have become prominent in thoracic surgery, including excessive central airway collapse, which includes EDAC and tracheobronchomalacia.^3^ Traditionally, surgery for TBP involves multiple horizontal sutures to secure a prosthesis to the cartilaginous wall of the trachea at each ring, along with plication of the membranous wall to restore normal anatomy and prevent excessive collapse. However, this approach can be time-consuming and may not ensure optimal prosthesis alignment in cases of EDAC. To address these challenges, we adopted a vertical suturing technique that reduces operative time and enhances prosthesis fit. The Heidelberg University Hospital's institutional ethics committee approved the collection and analysis of data from the patient's medical record (No. S-089/2018; January 3, 2018), and the requirement for individual informed patient consent for this retrospective study was waived.

## Surgical Technique

We present the case of a 39-year-old woman with long-standing asthma who was diagnosed with EDAC. After unsuccessful medical and endoscopic interventions, a multidisciplinary discussion determined that robotic-assisted TBP using a novel vertical suturing technique for prosthesis fixation was the most suitable option. The patient was positioned in the left lateral decubitus position with single-lung ventilation using a double-lumen endotracheal tube, and robotic-assisted TBP was performed using the Da Vinci X surgical system (Intuitive Surgical) (Video 1). Four 8-mm trocars were used: the camera port at seventh intercostal space (ICS) at the midaxillary line, second port at the eighth ICS anterior axillary, third at the eighth ICS posterior axillary, and fourth at the sixth ICS posterior scapular line, and a utility port at the ninth ICS between the camera and anterior ports; carbon dioxide insufflation was started after camera port placement. A 4-0 barbed suture (StrataFix Spiral PDS Plus Knotless Tissue Control Device; Ethicon) with a 17 mm, 1/2 circle taper point needle (RB-1, SXPP1B427; Ethicon) was used. Additionally, a monofilament polypropylene mesh (Bard Mesh; Becton, Dickinson, and Company) was selected for reconstruction. Special care was taken to avoid inadvertently suturing the endotracheal tube, which required periodic adjustment of its position throughout the procedure and partial-thickness bites. The prosthesis was custom-tailored intraoperatively using the robotic swabs as a reference, determining its length and width by comparing it to the swab size to ensure an optimal fit for the patient. The suturing began on the right side of the junction between the membranous and cartilaginous walls. This area was anchored to the mediastinal fatty tissue to maintain patency of the membranous wall and prevent prolapse. The suture line proceeded from left to right, advancing cranially to caudally, in a stepwise sequence involving the trachea, followed by the right main bronchus and subsequently the left main bronchus. Four suture rows were placed on the tracheal segment and 3 on the main bronchi. For the left main bronchus, suturing was performed with the endotracheal tube retracted, in combination with short periods of apnea, to allow for precise placement without tension or interference. The console time was 194 minutes and the skin-to-skin time was 223 minutes.

**Figure 1:**
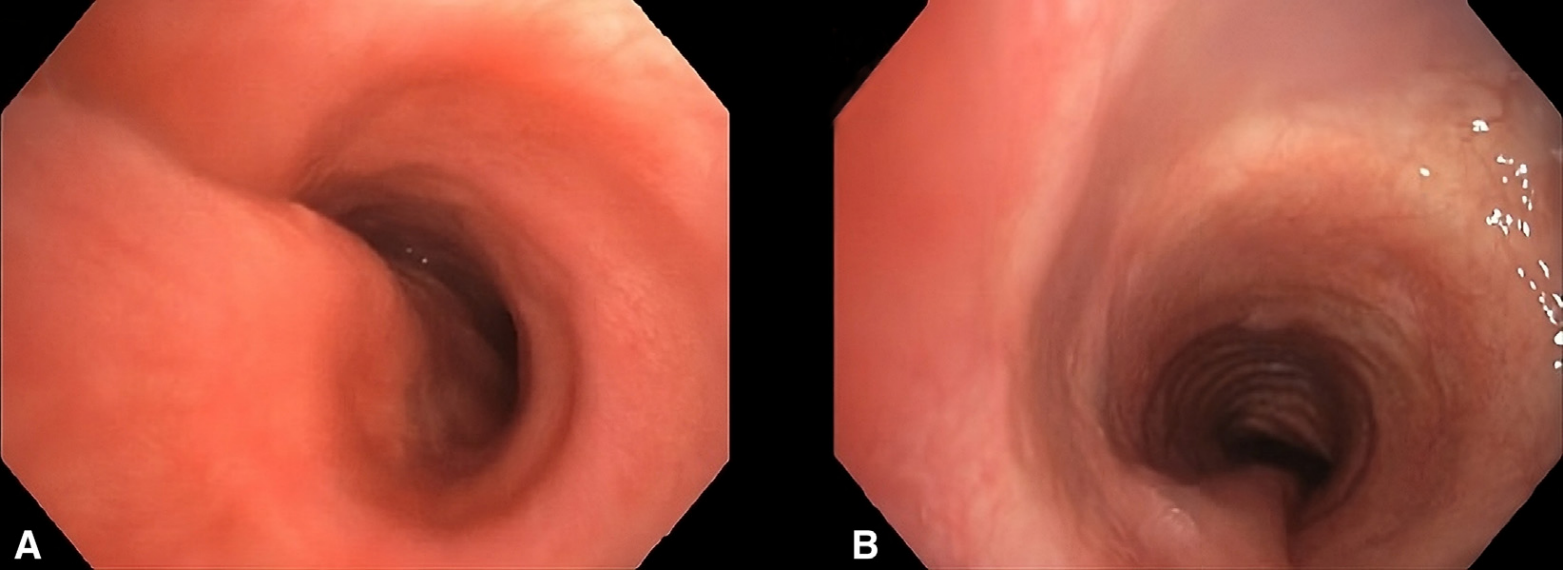
A, Preoperative bronchoscopic view showing significant collapse of the posterior tracheal wall. B, Postoperative view demonstrating a stable and open airway with no evidence of collapse.

On the seventh postoperative day, the patient underwent a follow-up bronchoscopy (Figure 1), which revealed no evidence of airway collapse, confirmed by a dynamic computed tomography scan (Video 2). Lung function testing, performed before discharge, demonstrated an improvement in forced expiratory volume in 1 second and forced vital capacity, indicating a positive influence on airflow dynamics postoperatively. Forced expiratory volume in 1 second increased from 0.94 L (27% predicted) preoperatively to 3.78 L (111% predicted) postoperatively, whereas forced vital capacity improved from 1.63 L (39% predicted) to 4.24 L (101% predicted).

## Discussion

Robotic-assisted TBP has emerged as a minimally invasive option that offers enhanced dexterity and precision, especially in confined spaces like the mediastinum.^3^ The Da Vinci system allows for superior visualization of the operative field and precise suture placement, making it well suited for procedures like TBP. In this case, the novel vertical suturing technique provided additional advantages, facilitating quicker suture placement by reducing the tissue manipulation that is often required with horizontal suturing. Vertical suturing may also promote more uniform tension distribution along the prosthesis, potentially enhancing the durability of the repair in cases of EDAC.

Particular attention was given to avoiding inadvertent suturing of the endotracheal tube, as described in the Surgical Technique section. The use of the robotic platform provided enhanced visualization and precision, facilitating periodic tube adjustments and the placement of partial-thickness bites with greater accuracy. This minimized the risk of airway compromise and underscores the advantage of robotic-assisted TBP in ensuring patient safety and procedural success, especially in delicate areas like the membranous wall.

Notably, the total operative time of 223 minutes in this case aligns with the existing literature on robotic-assisted TBP.^4^^,^^5^ This was the team's first case using this novel technique. Nevertheless, compared with TBP for tracheobronchomalacia, which often requires more complex tracheal narrowing, TBP for EDAC focuses on membranous wall reinforcement, potentially resulting in shorter operative times.

Nonetheless, vertical suturing raises questions regarding the long-term stability and durability of the repair compared with the more established horizontal suturing method. Horizontal suturing remains the standard in TBP, providing a robust framework for mesh placement along the length of the trachea and bronchi. Although vertical suturing using barbed sutures may reduce operative time and simplify the procedure, it lacks comprehensive follow-up data to validate its long-term effectiveness in maintaining airway patency and symptom relief.

## Conclusions

Robotic-assisted vertical suturing in tracheobronchoplasty for EDAC may reduce operative time, minimize tissue manipulation, and yield excellent short-term outcomes. However, long-term studies are needed to confirm its durability compared with traditional horizontal suturing.

## Conflict of Interest Statement

The authors reported no conflicts of interest.

The *Journal* policy requires editors and reviewers to disclose conflicts of interest and to decline handling or reviewing manuscripts for which they may have a conflict of interest. The editors and reviewers of this article have no conflicts of interest.

## Supplementary Data

Video 1 In the left lateral decubitus position with single-lung ventilation, 4 8-mm trocars and a utility port were placed with carbon dioxide insufflation. The posterior mediastinal pleura was opened to expose the trachea and mainstem bronchi. A prosthetic mesh was tailored to cover the weakened posterior membranous wall. Vertical sutures were placed initially through the mediastinal fatty tissue to widen the operative field and improve access to the membranous wall, followed by suturing from cranial to caudal ends along the trachea and continuing onto the main bronchi. Video available at: https://www.jtcvs.org/article/S2666-2507(25)00152-X/fulltext.

Video 2 Dynamic 3-dimensional computed tomography scan showing postoperative airway patency without collapse during expiration. Video available at: https://www.jtcvs.org/article/S2666-2507(25)00152-X/fulltext.
