# Supplementary figures and images for: Robotic tracheobronchoplasty with vertical suturing for excessive dynamic airway collapse
Source: JTCVS Tech. 2025 Apr 9;31:195–7. doi: 10.1016/j.xjtc.2025.03.024 (PMC12237877; doi:10.1016/j.xjtc.2025.03.024)

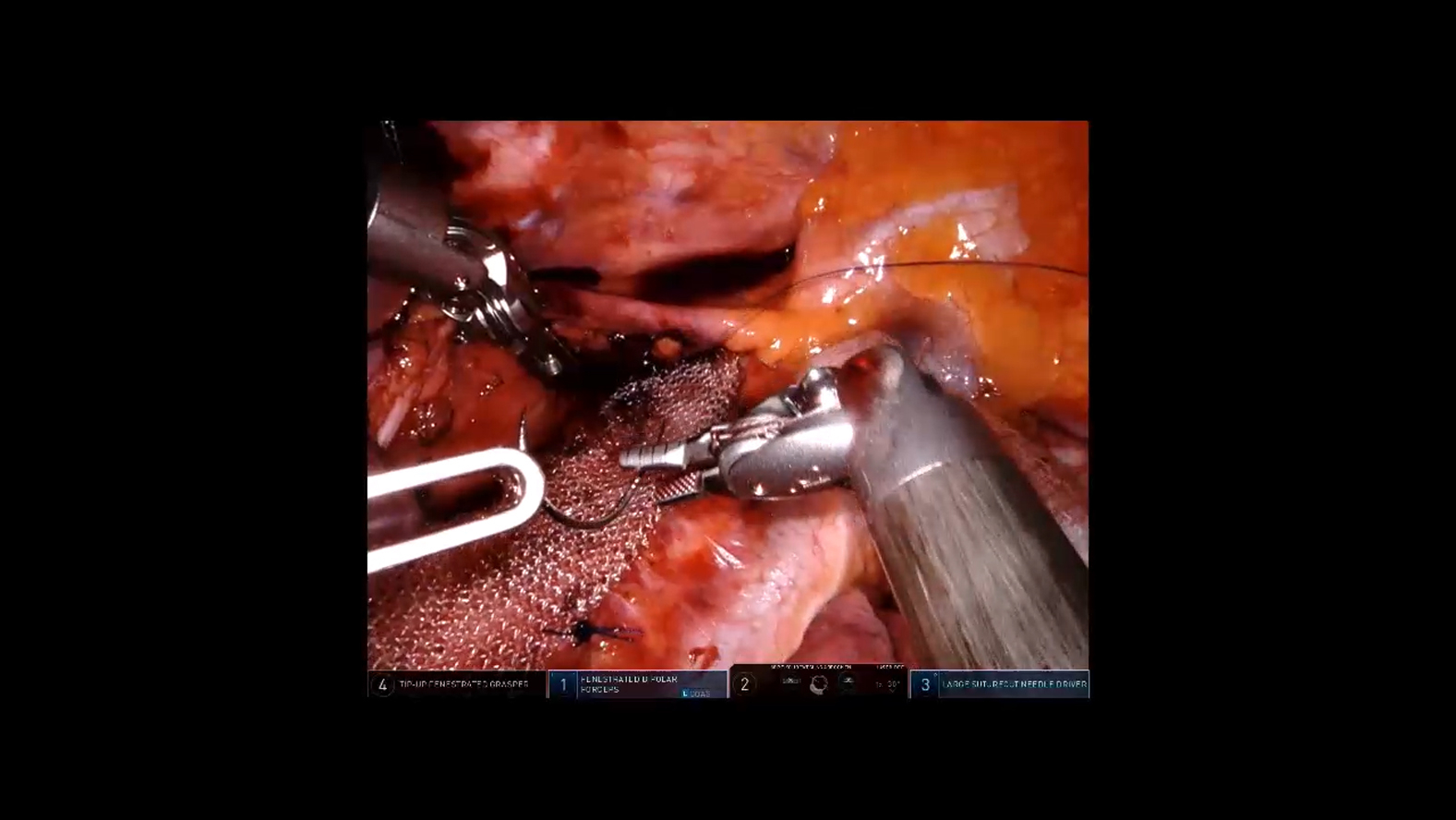

Supplement: Video 1 — In the left lateral decubitus position with single-lung ventilation, 4 8-mm trocars and a utility port were placed with carbon dioxide insufflation. The posterior mediastinal pleura was opened to expose the trachea and mainstem bronchi. A prosthetic mesh was tailored to cover the weakened posterior membranous wall. Vertical sutures were placed initially through the mediastinal fatty tissue to widen the operative field and improve access to the membranous wall, followed by suturing from cranial to caudal ends along the trachea and continuing onto the main bronchi. Video available at: https://www.jtcvs.org/article/S2666-2507(25)00152-X/fulltext. [file fx2.jpg]

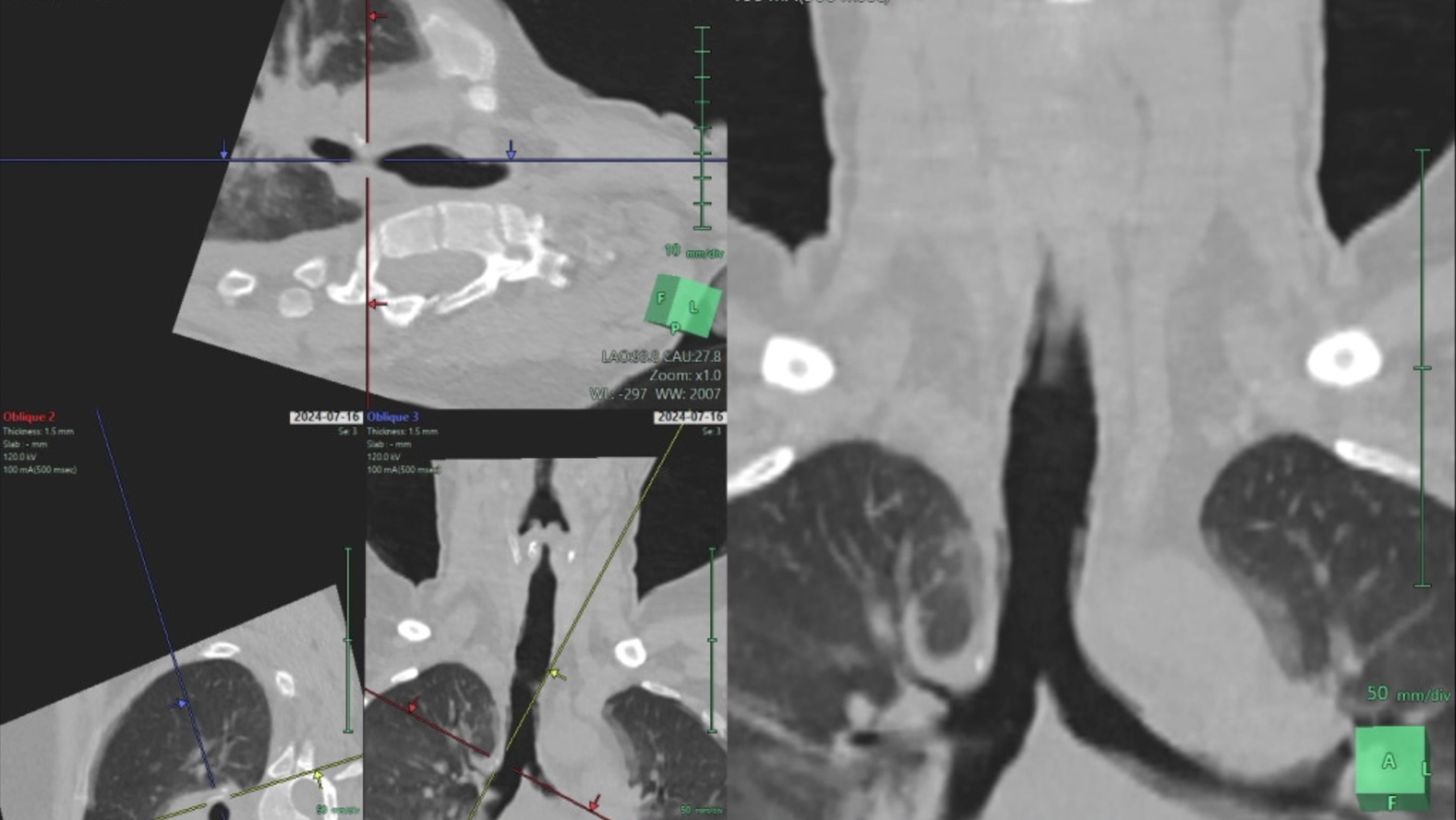

Supplement: Video 2 — Dynamic 3-dimensional computed tomography scan showing postoperative airway patency without collapse during expiration. Video available at: https://www.jtcvs.org/article/S2666-2507(25)00152-X/fulltext. [file fx3.jpg]
